# Acute flaccid myelitis associated with enterovirus-D68 infection in an otherwise healthy child

**DOI:** 10.1186/s12985-016-0678-0

**Published:** 2017-01-11

**Authors:** Susanna Esposito, Giovanna Chidini, Claudia Cinnante, Luisa Napolitano, Alberto Giannini, Leonardo Terranova, Hubert Niesters, Nicola Principi, Edoardo Calderini

**Affiliations:** 1 Pediatric Highly Intensive Care Unit, Department of Pathophysiology and Transplantation, Università degli Studi di Milano, Fondazione IRCCS Ca’ Granda Ospedale Maggiore Policlinico, Via Commenda 9, 20122 Milano, Italy; 2 Pediatric Intensive Care Unit, Fondazione IRCCS Ca’ Granda Ospedale Maggiore Policlinico, Milan, Italy; 3 Neuroadiology Unit, Department of Pathophysiology and Transplantation, Università degli Studi di Milano, Fondazione IRCCS Ca’ Granda Ospedale Maggiore Policlinico, Milan, Italy; 4 Department of Medical Microbiology, Division of Clinical Virology, University Medical Center Groningen, Groningen, The Netherlands

**Keywords:** Acute flaccid paralysis, Enterovirus D68, Neurological infection

## Abstract

**Background:**

Reporting new cases of enterovirus (EV)-D68-associated acute flaccid myelitis (AFM) is essential to understand how the virus causes neurological damage and to characterize EV-D68 strains associated with AFM.

**Case presentation:**

A previously healthy 4-year-old boy presented with sudden weakness and limited mobility in his left arm. Two days earlier, he had an upper respiratory illness with mild fever. At admission, his physical examination showed that the child was febrile (38.5 °C) and alert but had a stiff neck and weakness in his left arm, which was hypotonic and areflexic. Cerebrospinal fluid (CSF) examination showed a mild increase in white blood cell count (80/mm^3^, 41% neutrophils) and a slightly elevated protein concentration (76 gm/dL). Bacterial culture and molecular biology tests for detecting viral infection in CSF were negative. The patient was then treated with intravenous ceftriaxone and acyclovir. Despite therapy, within 24 h, the muscle weakness extended to all four limbs, which exhibited greatly reduced mobility. Due to his worsening clinical prognosis, the child was transferred to our Pediatric Intensive Care Unit; at admission he was diagnosed with acute flaccid paralysis of all four limbs. Brain magnetic resonance imaging (MRI) was negative, except for a focal signal alteration in the dorsal portion of the medulla oblongata, also involving the pontine tegmentum, whereas spine MRI showed an extensive signal alteration of the cervical and dorsal spinal cord reported as myelitis. Signal alteration was mainly localized in the central grey matter, most likely in the anterior horns. Molecular biology tests performed on nasopharyngeal aspirate and on bronchoalveolar lavage fluid were negative for bacteria but positive for EV-D68 clade B3. Plasmapheresis was performed and corticosteroids and intravenous immunoglobulins were administered. After 4 weeks of treatment, the signs and symptoms of AFM were significantly reduced, although some weakness and tingling remained in the patient’s four limbs. MRI acquired after 3 weeks showed that the previously reported alterations were no longer present.

**Conclusion:**

This case suggests that EV-D68 is a neurotropic agent that can cause AFM and strains are circulating in Europe. EV-D68 disease surveillance is required to better understand EV-D68 pathology and to compare various strains that cause AFM.

## Background

Until 2013, enterovirus (EV)-D68 was considered a rare cause of disease because only sporadic cases and minor outbreaks of respiratory infection occurred. However, since 2014, EV-D68 infection has gained epidemiological and clinical relevance when the virus caused a large-scale outbreak of severe respiratory infections, mainly in children, in the USA and Canada, with subsequent cases in other countries [1–3]. Respiratory disorders due to EV-D68 varied from pharyngitis and bronchitis to severe pneumonia with respiratory failure [4]. Additionally, concomitant with EV-D68-associated respiratory disease in the USA, there was an increase in the number of cases of severe neurological disease, mainly acute flaccid myelitis (AFM) [5]. EV-D68 was not detected in the cerebrospinal fluid (CSF) of these patients, and therefore definitive causation between EV-D68 and AFM was not established. However, the possibility of EV-D68 causing a polio-like disease was still considered. Other EVs, specifically EV-A71, are well known neurotropic agents that can cause aseptic meningitis, meningoencephalitis, and AFM [6]. Moreover, EV-D68 was detected in the respiratory secretions of several of the AFM cases diagnosed during the 2014 outbreak in the USA [5]. Finally, a case-control study of the presence of EV-D68 in upper respiratory secretions of children with acute respiratory infection demonstrated that children with infection and AFM were 10.3 times more likely to be infected with EV-D68 than children without AFM tested for respiratory infections [7]. Very few EV-D68-associated AFM cases have been reported in Europe [8–12]. Reporting new cases of this disease is essential to better understand how EV-D68 causes neurological damage and to identify and characterize the strains associated with AFM. In this paper, a case of AFM associated with EV-D68 infection occurring in an otherwise healthy child in Milan, Italy, is described.

## Case presentation

A previously healthy, fully immunized, 4-year-old boy came to the Emergency Room of the Saint Anna Hospital in Como on July 21, 2016, presenting with sudden onset weakness and poor mobility in his left arm. Two days earlier, he had an upper respiratory illness with mild fever, rhinorrhoea, and cough. Past and recent clinical histories were negative for relevant diseases. At admission, physical examination of the patient showed that the child had a temperature of 38.5 °C, a heart rate of 131/min, a respiratory rate of 18/min, 98% oxygen saturation and a blood pressure of 110/70 mmHg. Respiratory, cardiovascular, and skin examinations were normal. Neurological evaluation demonstrated that the child was alert but had a stiff neck and weakness in his left arm, which was hypotonic and areflexic. The results of cranial nerve examination, including fundoscopy, and diagnoses of the right arm and bilateral leg strength were normal.

Results from a complete blood count were in the normal range, with the exception of slightly elevated white blood cell and neutrophil counts. Serum electrolytes, coagulation factors, liver enzymes, and C-reactive protein were within the normal limits. Fine bilateral perihilar infiltration was observed on a chest X-ray to give the diagnosis of probable community-acquired pneumonia of viral aetiology.

CSF examination showed a mild increase in white blood cell count (80/mm^3^, 41% neutrophils) with normal glucose (60 mg/dL) and slightly elevated protein (76 gm/dL) concentrations. Cultures for bacteria were negative, and molecular biology tests for detecting viral infection were negative for herpes simplex virus 1, herpes simplex virus 2, human herpesvirus 6, EV, varicella zoster virus, adenovirus, parvovirus, cytomegalovirus, and Epstein-Barr virus. Computed tomography of the head without contrast did not show abnormalities. Intravenous ceftriaxone and acyclovir were then administered.

Despite this therapy, the child’s neurological function deteriorated rapidly. Within 24 h, the muscle weakness extended to all four limbs, which exhibited greatly reduced mobility. Due to his worsening clinical prognosis, the child was transferred to the Pediatric Intensive Care Unit (PICU) of the Fondazione IRCCS Ca’ Granda, Ospedale Maggiore Policlinico, Milan, Italy.

When the child was admitted to the PICU on July 22, 2016, he was alert but was diagnosed with acute flaccid paralysis based on neurological examination. Together with the muscles of the four limbs, the respiratory muscles were also clearly involved. Moreover, a deficit of the right facial nerve with dysphagia was observed. The child required intubation and mechanical ventilation for adequate gas exchange. After 11 days, tracheotomy was performed. Nutrition was assured through percutaneous endoscopic gastrotomy.

**Fig. 1:**
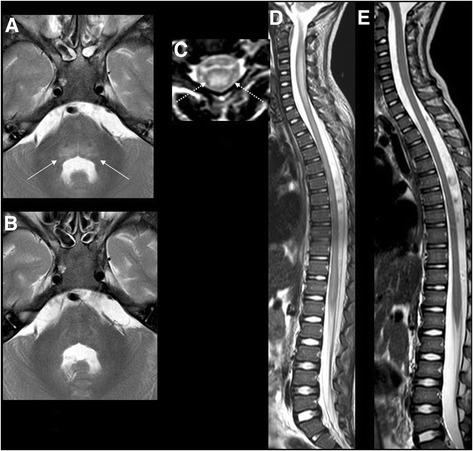
Magnetic resonance imaging (MRI) of the brain and spinal cord: **a**, axial T2 image at the level of the pons showing an increased signal in the tegmentum (*white arrows*), which was completely resolved in the follow up scan acquired 3 weeks later; **b**, sagittal and axial T2 image of the spinal cord (**c**, **d**) showing cord swelling, particularly at the cervical level (*white*, *dot arrow*), with extensive hyperintensity in the central cord (**d**), which was also completely resolved in the follow up images (**e**) acquired 3 weeks later

Figure 1a–d summarizes the results of magnetic resonance imaging (MRI) of the brain at admission to the PICU, 3 days and about 24 h after the onset of respiratory and neurological manifestations, respectively. A focal signal alteration was found localized in the dorsal portion of the medulla oblongata, also involving the pontine tegmentum, without enhancement or diffusion restriction. Moreover, MRI of the spinal cord showed an extensive signal alteration of the cervical and dorsal spinal cord, reported as myelitis. The signal alteration was mainly localized in the central grey matter, most likely in the anterior horns. After contrast administration, a slight enhancement in some of the caudal roots was observed, without enhancement in the spinal cord.

The presence of somatosensory-evoked potentials confirmed that only the motor pathway was affected. Acute polyradiculoneuritis was excluded because there was no albumino-cytological dissociation and no antiganglioside antibodies in the CSF. There was no evidence of paraneoplasic syndrome or inflammatory disease.

**Fig. 2:**
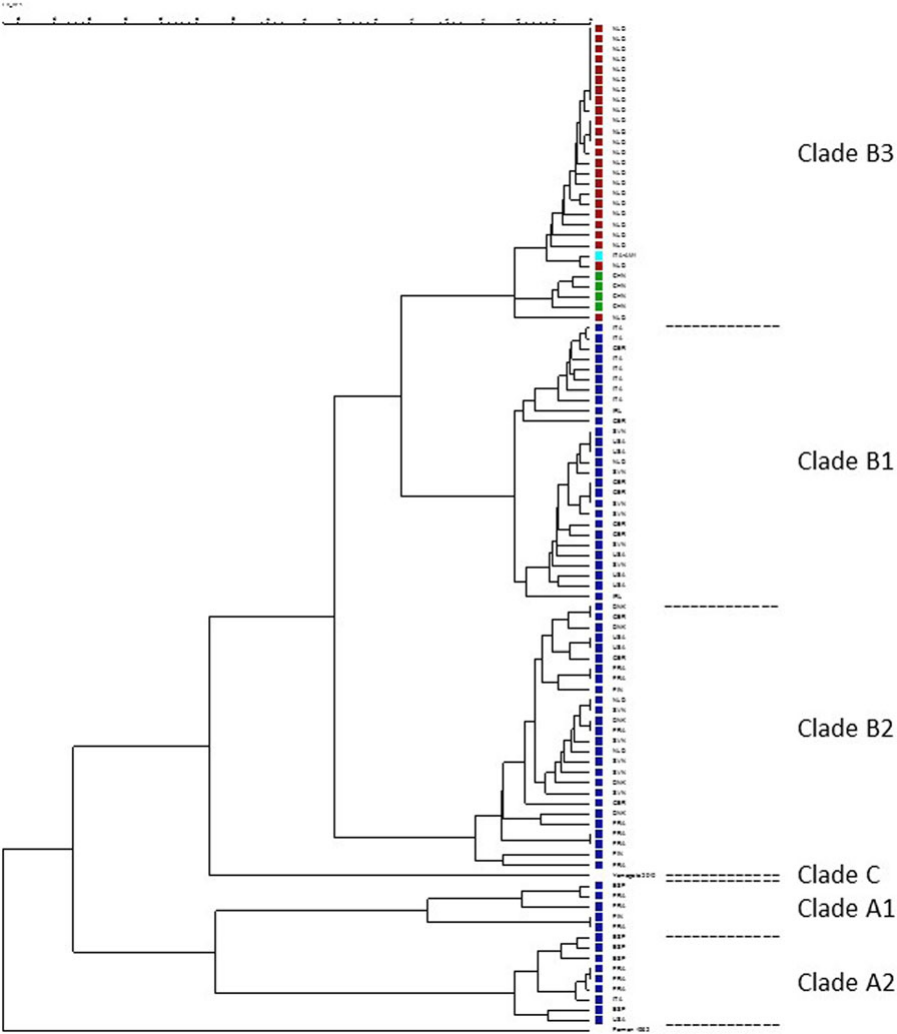
Phylogenetic tree: Sequence analysis showed that the strains obtained in 2016 (in *red*) are closely related to sequences of the recently described subclade B3, represented by four sequences from China (in *green*, 2014). The sequence from our Italian acute flaccid myelitis case (in *light blue*) is closely related to Dutch acute flaccid myelitis EV-D68 cases from 2016. Other 2014 cases are in dark blue. The nucleotide divergence was 2.1% within B3, 5.5% between B1 and B3, and 7.3% between B2 and B3. As outlier, the Fermon strain from 1962 was used. Analysis was performed using Bionumerics Software (Biomerieux, France) with sequencing partial VP1

Bacterial cultures of the nasopharyngeal aspirate and the bronchoalveolar lavage fluid were negative. Molecular biology tests for detecting viral infection were negative for herpes simplex virus 1, herpes simplex virus 2, human herpesvirus 6, varicella zoster virus, adenovirus, parvovirus, cytomegalovirus and EBV, but was positive for EV. Both the respiratory samples were reanalysed for the presence of EV-D68 using single-tube real-time PCR to amplify a 116 bp fragment of the 5′ non-translated region according to the method proposed by Poelman et al. [13]. Phylogenetic analysis based on the method used by Nix et al. [14] verified the presence of a virus belonging to clade B3 of the EV-D68 phylogenetic tree (Fig. 2). Sequence analysis showed that the 2016 strains were closely related to sequences of the recently described subclade B3, represented in Fig. 2, by four sequences obtained in China. The level of nucleotide divergence was 2.1% within B3, 5.5% between B1 and B3, and 7.3% between B2 and B3. The sequence from our Italian AFM case was closely related to Dutch AFM EV-D68 cases from 2016.

Serological analysis showed no infection with herpes simplex virus, varicella zoster virus, Epstein-Barr virus, cytomegalovirus, *Mycoplasma pneumoniae*, *Borrelia burgdorferi*, *Cryptococcus neoformans,* or *Mycobacterium tuberculosis*.

Treatment with intravenous methylprednisolone (30 mg/kg) was initiated. Plasmapheresis was conducted and intravenous immunoglobulins (1 g/kg/day) were administered during the first 3 days in the PICU. Intravenous steroid therapy was suspended after 5 days and substituted with oral prednisone (2 mg/kg/day) for 4 weeks, which was then tapered over an additional 2 weeks.

Significant weakness with reduced mobility of the four limbs and difficulty swallowing persisted with very slow regression. After 4 weeks of treatment, all the signs and symptoms of AFM were significantly reduced or disappeared, although a certain degree of weakness and tingling in the four extremities were still present. Moreover, deep tendon reflexes were generally reduced. However, as expected due to the recent onset of the disease, no muscle atrophy was observed. Moreover, the results of MRI performed about 1 month after the onset of the first neurological manifestations showed that the previously reported alterations were no longer present (Fig. 1e).

## Conclusions

AFM is a rare disease in polio-free geographical areas. Most of the cases are due to EVs, mainly EV-A71, flaviviruses, Japanese encephalitis virus, and West Nile virus [15, 16]. Recently, several AFM cases were diagnosed during an outbreak of EV-D68 respiratory infection, indicating an association between AFM and EV-D68 infection, although a direct causative role has not been established [3, 5]. However, EV-D68 has been detected in the cerebrospinal fluid of two patients with AFM [3, 5, 15, 17] and, more recently, in two other patients with aseptic meningitis [18].

The case described here suggests that EV-D68 is a neurotropic agent that can cause AFP. The case of AFM described here clinically resembles those described in the USA and Canada since 2014 [7] and the few cases described later in Europe [16]. AFM was diagnosed in a child suffering from a mild acute respiratory infection, who was febrile at the onset of neurological symptoms. Moreover, the pattern of neurological deficits and neuroimaging abnormalities localizing to the anterior horn cells of the spinal cord and cranial nerve motor nuclei in the brainstem are similar to those observed in other patients with AFM. Finally, CSF examination revealed alterations suggestive of aseptic meningitis. Together with these findings, the presence of EV-D68 in both the nasopharyngeal aspirate and BAL of the patient suggests a relationship between AFM and EV-D68, particularly because no other infection of the central nervous system could be found. The inability to detect EV-D68 in the CSF does not greatly weaken this relationship because an inability to detect an infectious agent in the central nervous system of patients with neurological complications from neurotropic viruses, including polio and EV-A71, is common [6]. A progressive reduction in signs and symptoms of disease occurred, albeit slowly, in the child described here, and MRI performed approximately 1 month after the onset of disease did not show persistent alterations. Although electromyogram and nerve conduction studies better correlate than MRI with final prognosis of AFM, this result suggests that this case could have a favourable prognosis, with long-term resolution of the neurological problems.

However, some reported cases have a less favourable outcome. Two children with EV-D68-associated AFM in Norway were recently described as having persistent deficits [8]. One had impaired head and motor control in his arm and the second developed atrophy in the left upper arm and shoulder. Thus, neurological damage due to EV-D68 infection can be severe and persistent, in a similar manner to polio infection and infection with other known neurotropic viruses.

The EV-D68 strain detected in this study was found to belong to the B3 clade. Despite all three clades of EV-D68 having been found to circulate worldwide, most of the cases detected in the USA [19] and Europe [3, 20] were from genetically similar viruses of clade B. Data regarding strains associated with the development of AFM are scarce. However, strains identified in the USA [16] were found to differ from other clade B strains in six amino acid substitutions, three of which are found in the structural and nonstructural proteins, thereby further defining the B1 clade. This finding led to the hypothesis that these strains could be significantly more neurotropic and virulent than other EV-D68 strains and that sequence data of the infecting strain could predict the clinical manifestations and outcomes of EV-D68 infection [21]. Our case report did not support this hypothesis and indicates that sequence data cannot predict clinical outcome. However, although characterization of EV-D68 is usually based on sequencing the VP1 region, mutations in other regions might confer higher virulence and increased risk of neurological complications during infection with certain EV-D68 strains. EV-D68 disease surveillance is warranted to clarify any potential differences in strain virulence and to define the pathogenic role of infection as a cause of AFM.

## Abbreviations

AFM: Acute flaccid myelitis
CSF: Cerebrospinal fluid
EV: Enterovirus
MRI: Magnetic resonance imaging
PICU: Pediatric intensive care unit
